# Magnetoelastic Coupling Evidence by Anisotropic Crossed Thermal Expansion in Magnetocaloric RSrCoFeO_6_ (R = Sm, Eu) Double Perovskites

**DOI:** 10.1021/acs.inorgchem.4c00594

**Published:** 2024-04-01

**Authors:** Romualdo S. Silva, João E. Rodrigues, Javier Gainza, Federico Serrano-Sánchez, Lidia Martínez, Yves Huttel, José Luis Martínez, José Antonio Alonso

**Affiliations:** † Instituto de Ciencia de Materiales de Madrid (ICMM), CSIC, E-28049 Madrid, Spain; ‡ European Synchrotron Radiation Facility (ESRF), 71 Avenue des Martyrs, 38000 Grenoble, France; § CELLS-ALBA Synchrotron Light Source, Cerdanyola del Vallès, E-08290 Barcelona, Spain

## Abstract

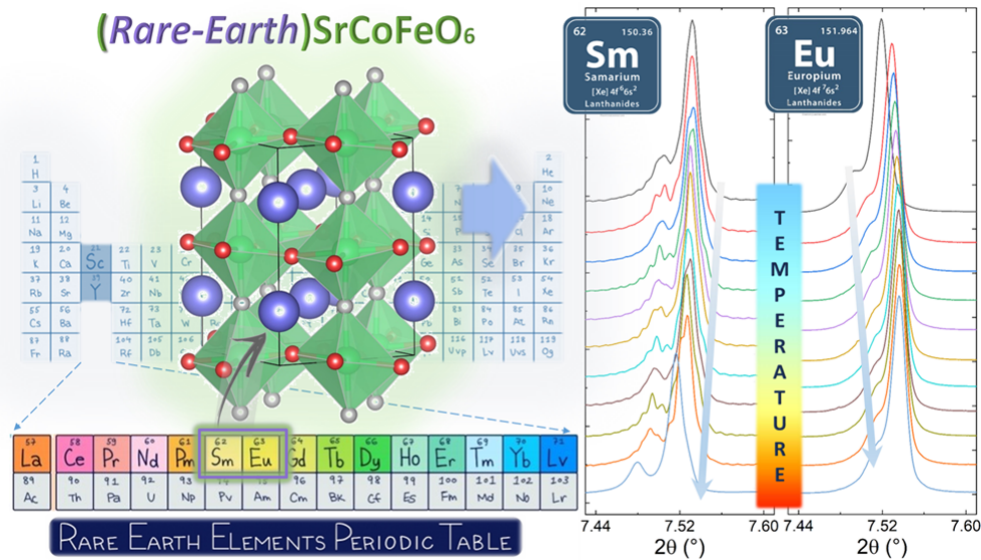

Double perovskite oxides, characterized by their tunable magnetic properties and robust interconnection between the lattice and magnetic degrees of freedom, present an enticing foundation for advanced magnetic refrigeration materials. Herein, we delve into the influence of rare-earth elements on RSrCoFeO_6_ (R = Sm, Eu) disordered double perovskites by examining their structural, electronic, magnetic, and magnetocaloric properties. Temperature-dependent synchrotron X-ray diffraction analysis confirmed the stability of the orthorhombic phase (*Pnma*) across a wide temperature range. X-ray photoemission spectroscopy revealed that both Sm and Eu are in the 3+ state, whereas multiple states for Co^2+/3+^ and Fe^3+/4+^ are identified. The magnetic investigation and magnetocaloric effect (MCE) analysis brought to light the presence of a long-range antiferromagnetic (AFM) order with a second-order phase transition (SOPT) in both samples. The maximum magnetic entropy change Δ*S*_M_^max^ was approximately 0.9 J/kg K for both samples at applied field 0–7 T, manifesting prominently above Neel temperatures *T*_N_ ≈ 93 K (Sm) and 84 K (Eu). Nevertheless, different relative cooling powers (RCP) of 112.6 J/kg (Sm) and 95.5 J/kg (Eu) were observed. A detailed analysis of the temperature-dependent lattice parameters shed light on a distinct magnetocaloric effect across the magnetic transition temperature, unveiling an anisotropic thermal expansion [α_V_ = 1.41 × 10^–5^ K^–1^ (Sm) and α_V_ = 1.54 × 10^–5^ K^–1^ (Eu)] wherein the thermal expansion axial ratio α_b_^Sm^/α_b_^Eu^ = 0.61 became lower with increasing temperature, which suggests that the Eu sample experiences a greater thermal expansion in the *b*-axis direction. At the atomic bonding level, the evidence for magnetoelastic coupling around the magnetic transition temperatures *T*_N_ was found through the anomalies along the average Co/Fe–O bond distance, formal valence, octahedral distortion, as well as an anisotropic lattice expansion.

## Abstract 2

The double perovskites RSrCoFeO_6_ (R = Sm, Eu), synthesized by solid-state procedures, contain R^3+^/Sr^2+^ and Co^2+/3+^/Fe^3+/4+^ ions randomly distributed on the two perovskite metal sites; from SXRD data, we observed anomalies in the lattice-parameter evolution, suggesting the occurrence of magnetoelastic coupling. The magnetocaloric effect analysis reveals substantial relative cooling powers (RCP), making them plausible candidates for cryogenic applications.

## 1 Introduction

With the global pursuit of sustainable and energy-efficient cooling solutions, magnetocaloric materials represent a fascinating class of materials that have attracted numerous research activities because of their intrinsic physical properties, which can contribute to magnetic refrigeration (MR) for specific applications. One of these properties relies on the magnetocaloric effect (MCE), a phenomenon in which a temperature variation in response to an applied magnetic field results in a magnetic entropy change (Δ*S*_M_).^1^ Other fundamental MCE insights are generally surrounded by many intrinsic and intriguing structural and magnetic features such as antisite disorder, magnetic frustration, and magnetoelastic coupling. In this perspective, the double perovskite (DP) oxides of formula (AA′)(BB′)O_6_ (AA′ = divalent and/or trivalent, BB′ = transition metals), with their tunable magnetic properties and strong coupling between lattice and magnetic degrees of freedom, offer a compelling platform for the development of magnetic refrigeration materials. These oxides can exhibit diverse magnetic behaviors, including ferromagnetic (FM), antiferromagnetic (AFM), or ferrimagnetic (FiM) ordering.^2−4^ The presence of multiple magnetic elements with different valence states and their interactions within the crystal lattice contribute to these varied magnetic properties. Understanding and harnessing the magnetocaloric effect in these compounds are essential steps for achieving breakthroughs in energy-efficient cooling systems.

Particularly, rare-earth (R)-based DP materials with highly localized 4f orbitals have been extensively investigated as possible candidates for MR through their MCE, essentially due to their large magnetic moment. For instance, R_2_CrMnO_6_ (R = Ho and Er),^5^ R_2_CuMnO_6_ (R = Gd, Dy, Ho, and Er),^6^ and R_2_FeCrO_6_ (R = Er and Tm)^7^ DP present a cryogenic MCE (i.e., MCE observed at *T* < 20 K) with maximum magnetic entropy change (Δ*S*_M_^max^) between 5.7 and 12.9 J/kg K under magnetic fields of 0 → 5–7 T. These compounds exhibit a disordered octahedral B-site and reveal a ferromagnetic–paramagnetic-like phase transition at ∼3–13 K, being attributed to their second-order phase transition (SOPT) nature. Additionally, the existence of the first-order phase transition (FOPT) becomes an unprecedented ingredient to enhance the MCE of these compounds, such as in the case of Er_2_CrFeO_6._^7^ In some cases, the FOPT can be promoted using a modest applied magnetic field (<7 T), which drives the order of a significant fraction of paramagnetic spins, leading to their maximized magnetocaloric properties. Still, the thermal and magnetic hystereses associated with FOPT lead to inefficiencies and rate limitations as well as greatly reduce the reversible adiabatic temperature change of magnetocaloric materials (a fact which is undesirable).^8^ To circumvent this situation, this work focuses on new disordered double-perovskite-based RSrCoFeO_6_ (R = Sm, Eu) oxides, which have the potential to lead to high magnetocaloric performance with enhanced magnetic entropy changes. In these compounds, the coupling behavior between magnetism and the crystal lattice plays a crucial role, the so-called magnetoelastic effect,^9−11^ manifested when magnetic ions interact with the crystal lattice structure in response to an external magnetic field, which is still poorly explored so far.

In this work, we investigate the influence of rare-earth elements in RSrCoFeO_6_ (R = Sm or Eu) oxides, focusing on their structural, electronic, magnetic, and magnetocaloric properties. Powders of the samples were synthesized by a solid-state reaction process. To perform the structural characterization, synchrotron X-ray diffraction (SXRD) patterns were collected in a wide temperature range to probe the evolution of the crystallographic structure. SXRD patterns in the 9–300 K range reveal an orthorhombic crystalline phase defined in the *Pnma* (no. 62) space group for both samples. Magnetic measurements of *M*(*T*) and *M*(*H*) as well as MCE analysis demonstrate the existence of a long-range AFM order for both samples with maximum magnetic entropy change Δ*S*_M_^max^ ≈ 0.9 J kg^–1^ K^–1^ (under a magnetic field of 0–7 T) above the Néel temperatures. The temperature-dependent lattice parameters derived from the Rietveld refinements suggested an anisotropic thermal expansion as well as a magnetoelastic coupling near the magnetic transition temperature. The valence states of the magnetic elements revealed from XPS analysis as Sm^3+^, Eu^3+^, Co^2+/3+^, and Fe^3+/4+^ were fundamental to interpreting the magnetism nature of the samples. Lastly, the cooling efficiency was determined through the relative cooling power (RCP), which follows the conventional power law and has been compared with other similar materials.

## 2 Experimental Section

### 2.1 Synthesis of Samples

The RSrCoFeO_6_ (R = Sm, Eu) samples were prepared by using the solid-state reaction process. First, the precursor oxides Sm_2_O_3_ (Alfa Aesar, 99.9% REO), Eu_2_O_3_ (Alfa Aesar, 99.9% REO), SrCO_3_ (Merck), Co_2_O_3_ (Merck, >99%), and Fe_2_O_3_ (Merck, >99%) were mixed in the proper stoichiometric ratio. Afterward, we placed the mixture together with 20 zirconia balls (∼5 mm diameter) in a Retsch PM100 planetary ball mill at 450 rpm for 30 min (dry without a medium). Finally, the powder was thermally treated for 12 h at 1100 °C in an air atmosphere, thus obtaining the final compounds.

### 2.2 Structural Characterization

SXRD data were recorded at the ID22 beamline at the ESRF (Grenoble, France) operating at a wavelength of λ = 0.35429 Å (= 35 keV).^12^ The RSrCoFeO_6_ (R = Sm, Eu) samples were sealed in a borosilicate capillary of 0.5 mm diameter and measured under rotation to minimize potential texture effects. The high-resolution powder diffraction patterns were collected over the range 1–40° (2θ) in the continuous scanning mode for temperatures ranging from 9 to 300 K with a ∼1 min waiting time at each temperature step to guarantee an isothermal condition. SXRD patterns were retrieved following the processing method described in ref (13). They were analyzed by Rietveld refinement using the FullProf program.^14^ The peak shape was described with a pseudo-Voigt function, and the background was interpolated between areas devoid of reflections. The full refinement included the following parameters: scale factors, zero-point error, background coefficients, asymmetry correction factors, lattice parameters, atomic positions, occupancy factors, and isotropic displacement parameters.

### 2.3 Surface Chemistry and Electronic Structure

X-ray photoelectron spectroscopy (XPS) was carried out in a chamber with a base pressure of 10^–10^ mbar using a hemispherical electron energy analyzer (SPECS Phoibos 100 spectrometer) and an X-ray source at Al Kα (1486.29 eV) operated at 150 W. The powder samples were deposited onto clean and conductive double-sided carbon tape, loaded in a vacuum load-lock chamber, and finally transferred to ultrahigh vacuum. No cleaning protocol with argon bombardment was considered in order to avoid Ar^+^-induced electronic changes (in particular, preferential sputtering in oxides). The angle between the hemispherical analyzer and the plane of the surface was kept at 60°. The survey spectra were recorded with a step of 0.5 eV and a pass energy of 40 eV. Specific core-level spectra (Sm 3*d*, Eu 3*d*, Sr 3*d*, Co 2*p*, Fe 2*p*, O 1*s*, and C 1*s*) were acquired with an energy step of 0.1 eV and a pass energy of 20 eV. Data processing was performed within the *CasaXPS* software (Casa software Ltd., Cheshire, UK), and the absolute binding energies were adjusted to the binding energy of the C 1*s* core level at 285 eV.^15^ Peak areas were obtained by fitting the spectra and using the relative sensitivity factors from the atomic photoionization cross section of each core level, provided by the SPECS Prodigy library. In all of the fittings, we constrain the peak positions and widths according to the respective multiplet theory, and the spectra were normalized for easier comparison.

### 2.4 Morphological and Elemental Analyses

Field emission scanning electron microscopy (FE-SEM) images and energy-dispersive X-ray (EDX) analysis were taken by using an FEI Nova NanoSEM 230 microscope complemented with an Apollo XL Silicon Drift detector (SDD) from EDAX-Ametek. Powders of the samples were stuck to carbon adhesive tape and visualized without a conductive coating.

### 2.5 Magnetic Measurements

The magnetic properties were measured in a SQUID magnetometer (MPMS-3), from Quantum Design (San Diego, USA), at temperatures ranging from 1.8 up to 300 K and applied magnetic fields up to 7 T. The *M*(*H*) curves for the magnetocaloric effect analysis were collected with temperature intervals of Δ*T* = 3 K and applied field up to 7 T.

## 3 Results

### 3.1 Room-Temperature Crystallographic Structure

**Figure 1:**
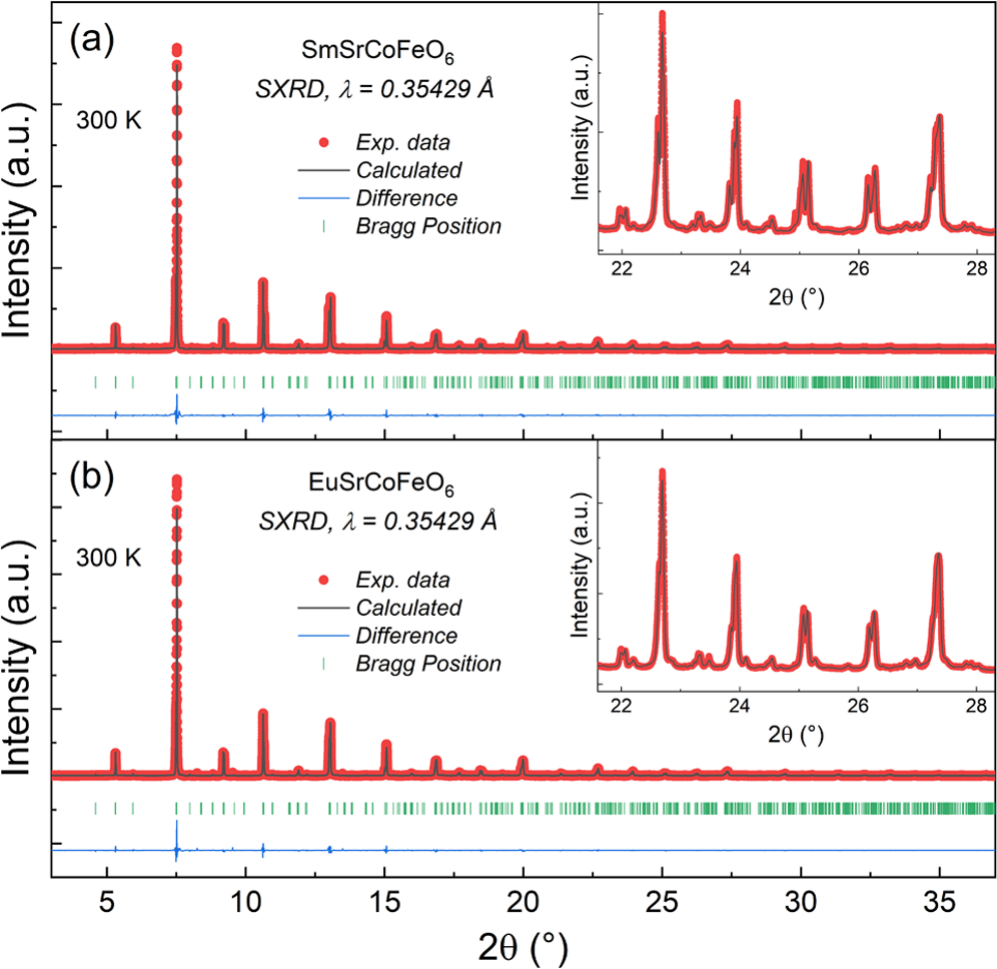
Rietveld refinement from SXRD data for (a) SmSrCoFeO_6_ and (b) EuSrCoFeO_6_ oxides collected at room temperature. The inset displays the profile refinement for high diffraction angles between ∼21 and 28°. Raw data are represented as red symbols, while the corresponding fit from Rietveld refinement is drawn as black solid lines. The blue curve at the bottom represents the fit residual, while the green lines correspond to theoretical expected Bragg diffraction peaks.

In Figure 1, the SXRD patterns at room temperature together with their best Rietveld refinement for the RSrCoFeO_6_ (R = Sm, Eu) samples are shown, which reveal an orthorhombic crystalline phase belonging to the *Pnma* (no. 62) space group for both samples. In the inset, the profile fitting quality for high diffraction angles confirms this assumption. The obtained lattice parameters were *a* = 5.3984(7) Å, *b* = 7.6334(9) Å, *c* = 5.4329(3) Å, and *V* = 223.88(7) Å^3^; and *a* = 5.3985(1) Å, *b* = 7.6329(8) Å, *c* = 5.4235(7) Å, and *V* = 223.48(7) Å^3^ for both Sm and Eu samples, respectively. The refined parameters for both samples at room temperature are given in Table S1 in the Supporting Information. The Co/Fe–O average bond lengths of 1.918 Å (Sm) and 1.920 Å (Eu) are comparable to or slightly lower than those of GdSrCoFeO_6_ (1.927 Å)^11^ and NdSrCoFeO_6_ (1.938 Å);^16^ hence, it is reasonable to conclude that Fe^3+^ is in the high-spin (HS) state, whereas Co^3+^ can manifest an HS to an intermediate-spin (IS) state transition in the octahedral environment,^16^ but further confirmation is needed using, for instance, X-ray emission spectroscopy.^17^ From the ⟨Co/Fe–O–Co/Fe⟩ average bond angles of 171.9° (Sm) and 168.8° (Eu), the octahedral tilting can be calculated through the expression Φ = [180 – ⟨Co/Fe–O–Co/Fe⟩]/2,^18^ resulting in Φ = 4.1 and 5.6°, respectively, which indicates a slight octahedral tilting in both samples. Besides, the intensity of the small peak before 2θ ≈ 5° is correlated to the degree of B-site disorder in these crystalline structures.^19−21^ Thus, from the SXRD data (see Figure S1), we can conclude that the Eu sample presents a higher degree of Co/Fe-site long-range structural disorder.

### 3.2 Temperature-Dependent Crystalline Structure

**Figure 2:**
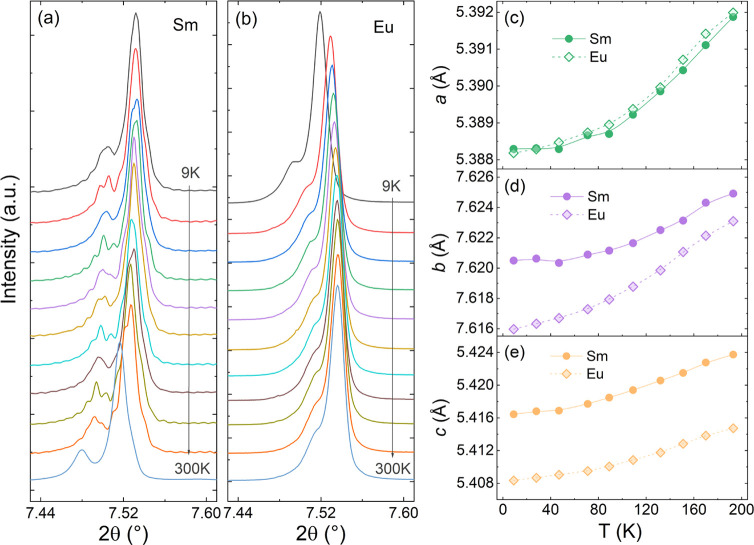
Temperature-dependent shift of SXRD peaks for the RSrCoFeO_6_ samples: (a) Sm and (b) Eu. (c–e) Thermal evolution of lattice parameters *a*, *b*, and *c*, respectively.

We investigate the thermal evolution of the crystal structure by temperature-dependent SXRD data in the 9–193 K range (raw patterns are represented in Figure S2). All of the SXRD data were refined with *Pnma* symmetry, ruling out any global structural phase transition down to 9 K for both samples (see Figure S3). It is noteworthy that the main SXRD peak position moves toward lower 2θ angles with increasing temperature in the Sm sample, whereas for the Eu sample it moves to higher 2θ angles (see Figures 2a,b and S4). According to Bragg’s law [*n*λ = 2*d *sin(θ)], larger diffraction angles would result in smaller interatomic distances between crystallographic planes, and vice versa.^22^ In the case of the Eu sample, we would expect a decrease in the lattice parameters leading to a negative thermal expansion, such as in Pb_2_CoMoO_6_^23^ or Cu_2_PVO_7_.^24^ The temperature-dependent lattice parameters derived from the Rietveld refinement analysis are displayed in Figure 2c–e. The calculated thermal expansion coefficients for the lattice parameters were (Sm) 3.6 × 10^–6^ K^–1^/(Eu) 3.8 × 10^–6^ K^–1^ for the *a*-axis, (Sm) 3.15 × 10^–6^ K^–1^/(Eu) 5.14 × 10^–6^ K^–1^ for the *b*-axis, and (Sm) 7.34 × 10^–6^ K^–1^/(Eu) 6.33 × 10^–6^ K^–1^ for the *c*-axis in the same temperature range.

It is worth mentioning that the thermal expansion axial ratio between Sm and Eu samples of α_b_^Sm^/α_b_^Eu^ = 0.61 became lower at increased temperatures (at 9–193 K range) compared to α_a_^Sm^/α_a_^Eu^ = 0.95 and α_c_^Sm^/α_c_^Eu^ = 1.16, suggesting that the Eu sample experiences a greater thermal expansion in the *b*-axis direction (see Figure 2d), which can be associated with an anisotropic thermal expansion of the orthorhombic unit-cell. This observation may explain the difference in the shift direction of the SXRD peaks compared to the Sm sample (see Figure S4). A similar anisotropic thermal expansion feature was recently observed for Sr_2–*x*_La_*x*_CoNbO_6._^25^

### 3.3 Morphology and Chemical Composition

**Figure 3:**
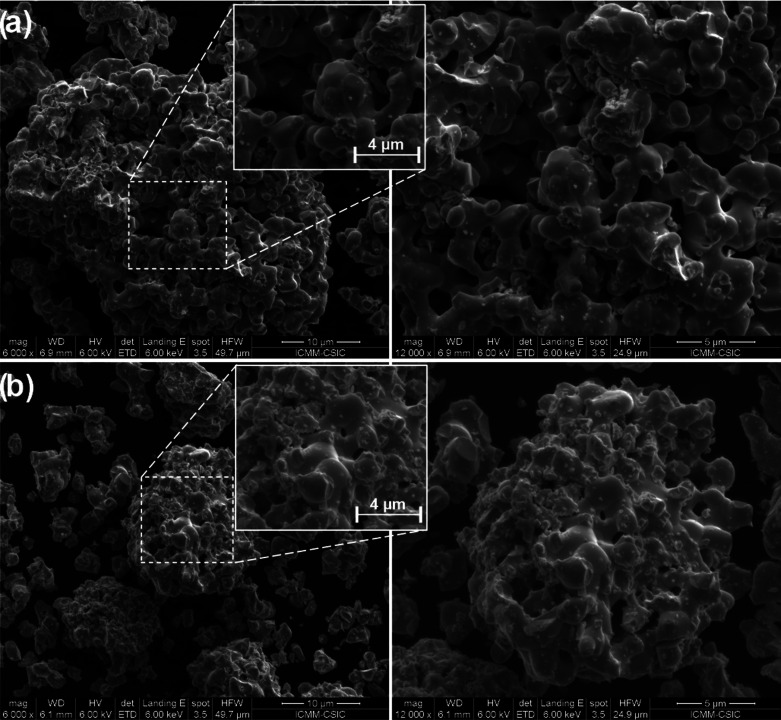
FE-SEM images of (a) SmSrCoFeO_6_ and (b) EuSrCoFeO_6_ samples at (left) × 6000 and (right) × 12 000 magnification.

The morphological characterization of the RSrCoFeO_6_ (R = Sm, Eu) samples was performed by FE-SEM, and their respective images are given in Figure 3. The FE-SEM images obtained demonstrate that the synthesized powders consist of typical polycrystalline structures with irregularly shaped grains and nonuniform size distribution in both samples. Also, it is notable that the samples present a large range of grain sizes (1–10 μm), with more elongated particle shapes for SmSrCoFeO_6_ and smaller spherical shapes for EuSrCoFeO_6_. In this regard, the grain arrangements and their relative size distribution are a consequence of the different rates and natures of the nucleation process for each of the samples.

To confirm the atomic composition of the samples, EDX analysis was performed as illustrated in Figure S5. No trace of impurity peaks was detected in the EDX spectra, which confirmed the purity and homogeneity of the samples. Moreover, the elemental analysis for Sm, Eu, Sr, Co, and Fe composition is consistent with the ideal stoichiometric values (within the tolerance limit), yielding ratios of Sm/Sr ≈ 0.91 and Co/Fe ≈ 1.03 for SmSrCoFeO_6_ and Eu/Sr ≈ 1.22 and Co/Fe ≈ 0.95 for EuSrCoFeO_6_. Indeed, the [Sm + Sr]/[Co + Fe] ≈ 1.08 and [Eu + Sr]/[Co + Fe] ≈ 0.94 ratios indicate that the SmSrCoFeO_6_ sample presents a lower relative amount of Co/Fe ions compared to that of EuSrCoFeO_6_. On the other hand, oxygen vacancies were observed for both samples, being greater for EuSrCoFeO_6_.

### 3.4 Electronic Structure

**Figure 4:**
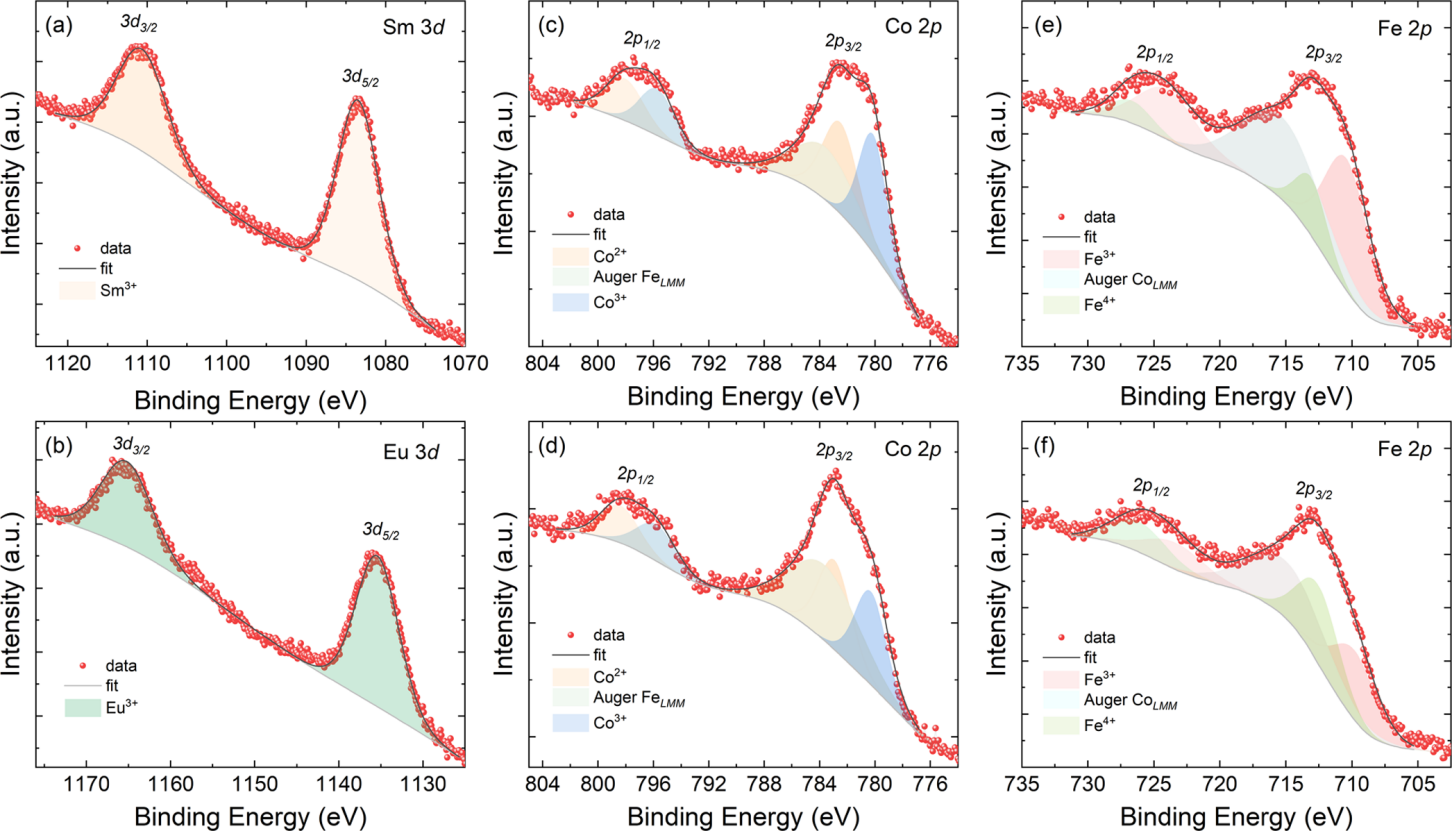
X-ray photoemission spectra (XPS) of Sm 3*d*, Eu 3*d*, Co 2*p*, and Fe 2*p* levels for SmSrCoFeO_6_ (a,c,e) and EuSrCoFeO_6_ (b,d,f) samples.

Core-level XPS provides insights into the charge state of the atoms forming the lattice. Herein, we were mainly interested in analyzing the spectra of the magnetic elements. Thus, a detailed analysis of the Sm 3*d*, Eu 3*d*, Co 2*p*, and Fe 2*p* core-level XPS spectra is presented in Figure 4 for the SmSrCoFeO_6_ and EuSrCoFeO_6_ samples. For simplicity, the BE reported for Sm, Eu, and Sr corresponds to the 3*d*_5/2_ emission, while that for Fe and Co is the 2*p*_3/2_ emission. The core-level XPS spectrum for Sm 3*d* in Figure 4a reveals a component at 1083.5 eV (Sm 3*d*_5/2_),^26^ indicating that all samarium detected is in the form of Sm^3+^ at the surface of SmSrFeCoO_6_. Similarly, the core-level XPS spectrum for Eu 3*d* in Figure 4b presents a peak at 1135 ± 0.5 eV (Eu 3*d*_5/2_), which can be assigned to Eu^3+^^27^ at the surface of EuSrCoFeO_6_.

The analysis of Co 2*p* in both samples (Figure 4c,d) revealed the presence of two components, one at 780.2 ± 0.1 eV, which can be attributed to Co^3+^, whereas the other component at 798.2 ± 0.2 eV can be ascribed to Co^2+^^65^. There is an additional component needed for the correct fitting of the spectra at 783.6 eV, which corresponds to the Auger Fe_LMM_.^28^ Likewise, the analysis of the Fe 2*p* core-level spectrum (Figure 4e,f) reveals the presence of a first peak at 724.7 ± 0.3 eV that corresponds to Fe^3+^^29,30^ and a second component at 726.4 ±0.3 eV usually associated with Fe^4+^.^31,32^ As occurred in Co 2*p*, the presence of an additional peak around 715 eV is related to the presence of the Auger Co_LMM_ peak.^33^

In the case of Sr 3*d* (Figure S6c,d), it was observed a component at 132.5 ± 0.5 eV, which can be attributed to Sr^2+^ in the lattice.^34^ In both samples, additional peaks were needed to fit the spectra, caused by the overlapping with Sm 4*d* (136.6 eV) and Eu 4*d* (136.5 eV)^35^ in a significant proportion (Sm/Sr ≈ 0.5 and Eu/Sr ≈ 1.5). Finally, the O 1*s* core-level spectra exhibit four components (see Figure S6e,f). The peak at lower BE (528.9 ± 0.1 eV) is attributed to lattice oxygen species, O^2–^.^36^ The peak at 531.5 ± 0.1 eV is usually attributed to the contributions of [Co/Fe]O_6_ octahedra cations and surface species in the termination layer.^37^ At higher BE, the two components around 533.0 ± 0.1 and 534 ± 0.1 eV can be related to the presence of chemisorbed species, either O or OH^–^ species.^38^ These results are in good agreement with other similar double perovskites.^16,39^ The quantitative analysis of elements is summarized in Table S2.

### 3.5 Magnetic Properties

**Figure 5:**
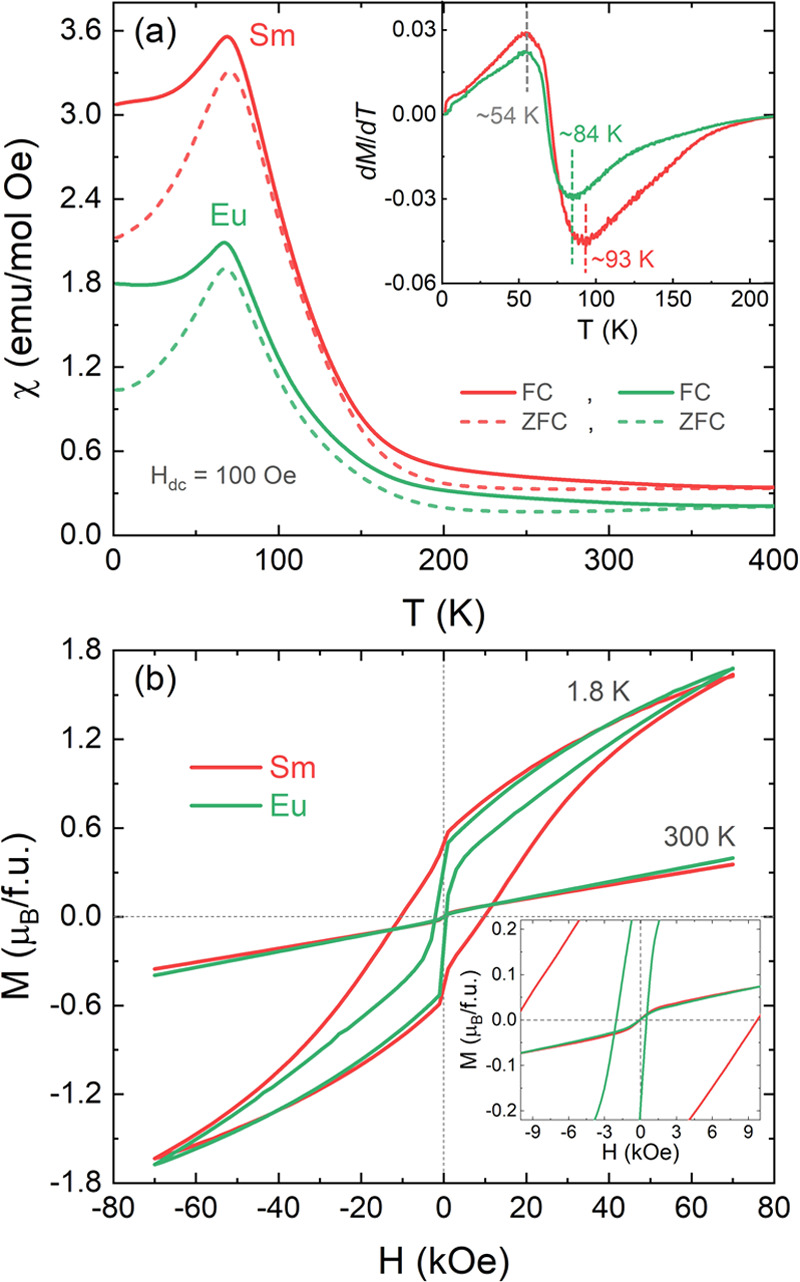
(a) Temperature-dependent susceptibility (χ = *M*/*H*) of RSrCoFeO_6_ (R = Sm, Eu) samples measured at an applied field of *H*_dc_ = 100 Oe in the zero-field-cooled (ZFC) and field-cooled (FC) protocols. In the inset, the magnetization-temperature derivative (d*M*/d*T*) versus temperature (*T*) curves are shown. (b) *M*(*H*) isotherms at 1.8 and 300 K for an applied field up to 70 kOe. The inset shows a zoom for the low-field region.

The temperature-dependent d*c* magnetic susceptibilities χ(*T*) in both zero-field-cooled (ZFC) and field-cooled (FC) modes are represented in Figure 5a. Typical profiles with cusp peaks around the magnetic transition suggest the existence of long-range AFM order at the Néel temperatures *T*_N_ ≈ 93 K (Sm) and 84 K (Eu) [highlighted on minima of d*M*/d*T*(*T*) curves; see the inset]. The higher *T*_N_ for the Sm sample is supported by the increased Co/Fe-site order compared to that for the Eu sample (as explained in the SXRD analysis). Moreover, the slightly larger ionic radius of the Sm^3+^ (1.079 Å, coordination number = VIII) ion compared to Eu^3+^ (1.066 Å, coordination number = VIII)^40^ leads to lower octahedral tilting for the Sm sample, which then favors enhanced long-range magnetic ordering due to the dominating Co–O–Fe superexchange interactions.

In particular, the temperature around 54 K (same for both samples) in the maximum of d*M*/d*T*(*T*) curves is probably related to the polarization of paramagnetic (PM) rare-earth ions (Sm and Eu) under an applied magnetic field, which determines the magnetic order transition range toward the PM state (above *T*_N_). The divergence of ZFC/FC curves is attributed to magnetic irreversibility due to magnetic frustration, i.e., the FM components of Co/Fe ions randomly couple with the AFM matrix at low temperatures.^16^ These findings were further supported by the *M*(*H*) isotherm curves (see Figure 5b), which exhibit a notable hysteresis loop at 1.8 K with coercive fields *H*_C_ ≈ 10 kOe (Sm) and 1 kOe (Eu), as well as a nonsaturated magnetization of ∼1.7 μ_B_/f.u (in both samples at 70 kOe). This suggests that the Sm sample has a larger FM component compared with the Eu sample. The *M*(*H*) curves at 300 K do not show a completely linear isotherm behavior at low fields (see inset of Figure 5b) due to the remaining regions whose spins are still polarized with the magnetic field.

### 3.6 Magnetocaloric Performance

**Figure 6:**
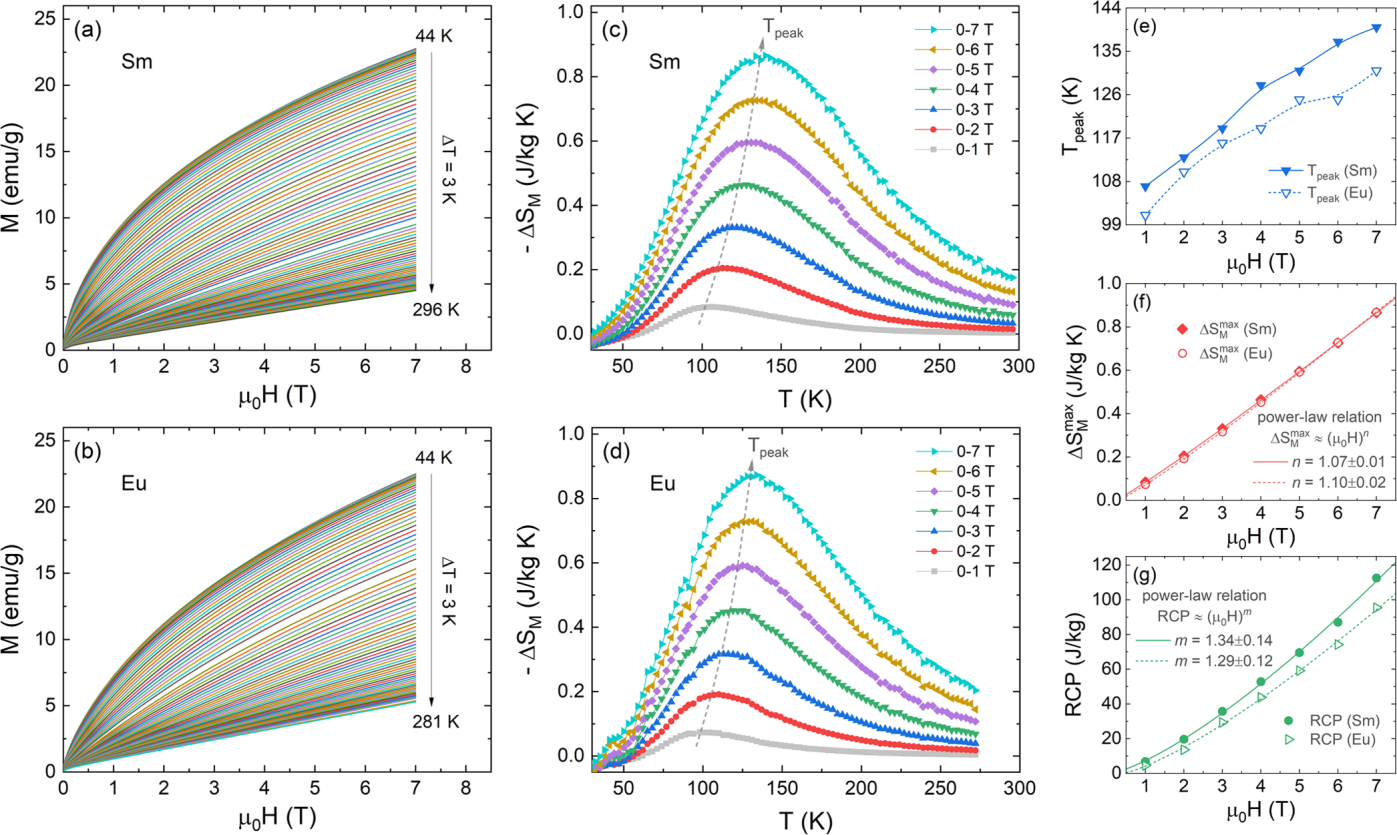
(a, b) Isothermal raw magnetization *M*(*H*) curves at different temperatures of 44 up to 296 K under an applied magnetic field μ_0_*H* up to 7 T. (c, d) −Δ*S*_*M*_(*T*) curves at magnetic fields from 0–1 to 0–7 T obtained by the isotherm raw data. (e) *T*_peak_ variation as a function of μ_0_*H* for the Sm and Eu samples. (f, g) Δ*S*_M_^max^ and RCP^max^ behavior dependent on μ_0_*H* and their respective power-law fittings.

To investigate the magnetocaloric effect of Sm- and Eu-based DP samples, we performed isothermal magnetization *M*(*H*) measurements at several temperatures, as shown in Figure 6a,b. As noted, the *M*(*H*) curves underwent a continuous thermomagnetic transition from a weak FM/AFM order (*T* < *T*_N_) to a PM state (*T* > *T*_N_). To further understand the ordering of the magnetic phase transition, Arrott plots (*M*^2^ vs *H*/*M*) were represented from the *M*(*H*) data in the same temperature range, as displayed in Figure S7. According to Banerjee’s criterion,^41^ the general behavior of the curves indicates a second-order phase transition (SOPT) for both samples, similar to other double perovskites.^16,39^

The magnetocaloric effect through the magnetic entropy change (Δ*S*_M_) was determined from *M*(*H*) raw isotherm measurements by numerical integration of Maxwell’s thermodynamic relation,^42^ as given by
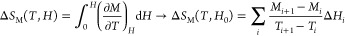
1where *M*_*i*_ and *M*_*i+1*_ are the magnetizations obtained at temperatures *T*_*i*_ and *T*_*i+1*_, under a magnetic field *H*_*i*_, respectively.

In Figure 6c,d, the temperature dependence of magnetic entropy change Δ*S*_M_(*T*) for applied fields from 1 up to 7 T are plotted for the Sm and Eu samples, respectively. Δ*S*_M_(*T*) curves reveal a conventional MCE behavior with well-defined positive peaks for both samples. The maximum value of Δ*S*_M_ [Δ*S*_M_^max^ at *T*_peak_] was found to be around the magnetic phase transition temperature, which is shifted toward higher temperatures when the applied field increases up to 7 T (see Figure 6e). The Δ*S*_M_^max^ follows the power law Δ*S*_M_^max^ ≈ (μ_0_*H*)^*n*^ as demonstrated in Figure 4f, yielding exponents *n* = 1.07 ± 0.01 (Sm) and 1.10 ± 0.01 (Eu). These *n* values are greater than those of mean-field ferromagnets (*n* = 0.67),^43^ which is another evidence of the presence of a weak FM/AFM state in both samples. Particularly, at μ_0_Δ*H* = 0–7 T, the Δ*S*_M_^max^ value for both samples is practically the same, ∼0.87 J/kg K, and lower compared to GdSrCoFeO_6_ (∼13 J/kg K).^39^ On the other hand, this value is close to that observed for other double perovskites, such as Sm_2_CoMnO_6_ (1.4 J/kg K at 0–6 T)^44^ and Eu_2_NiMnO_6_ (3.2 J/kg K at 0–5 T).^2^

## 4 Discussion

Based on the results presented above, we link the structural properties derived from state-of-the-art synchrotron X-ray diffraction and the electronic properties from XPS analysis with the magnetic properties exhibited by RSrCoFeO_6_ (R = Sm, Eu) samples. We start by evaluating the thermal expansion of the unit cells for both compounds followed by the evidence of magnetoelastic coupling around the magnetic transition temperature. From the XPS and magnetic measurements, the rare-earth magnetic moments, frustration, and magnetic refrigeration are then discussed.

### 4.1 Volumetric Thermal Variation

**Figure 7:**
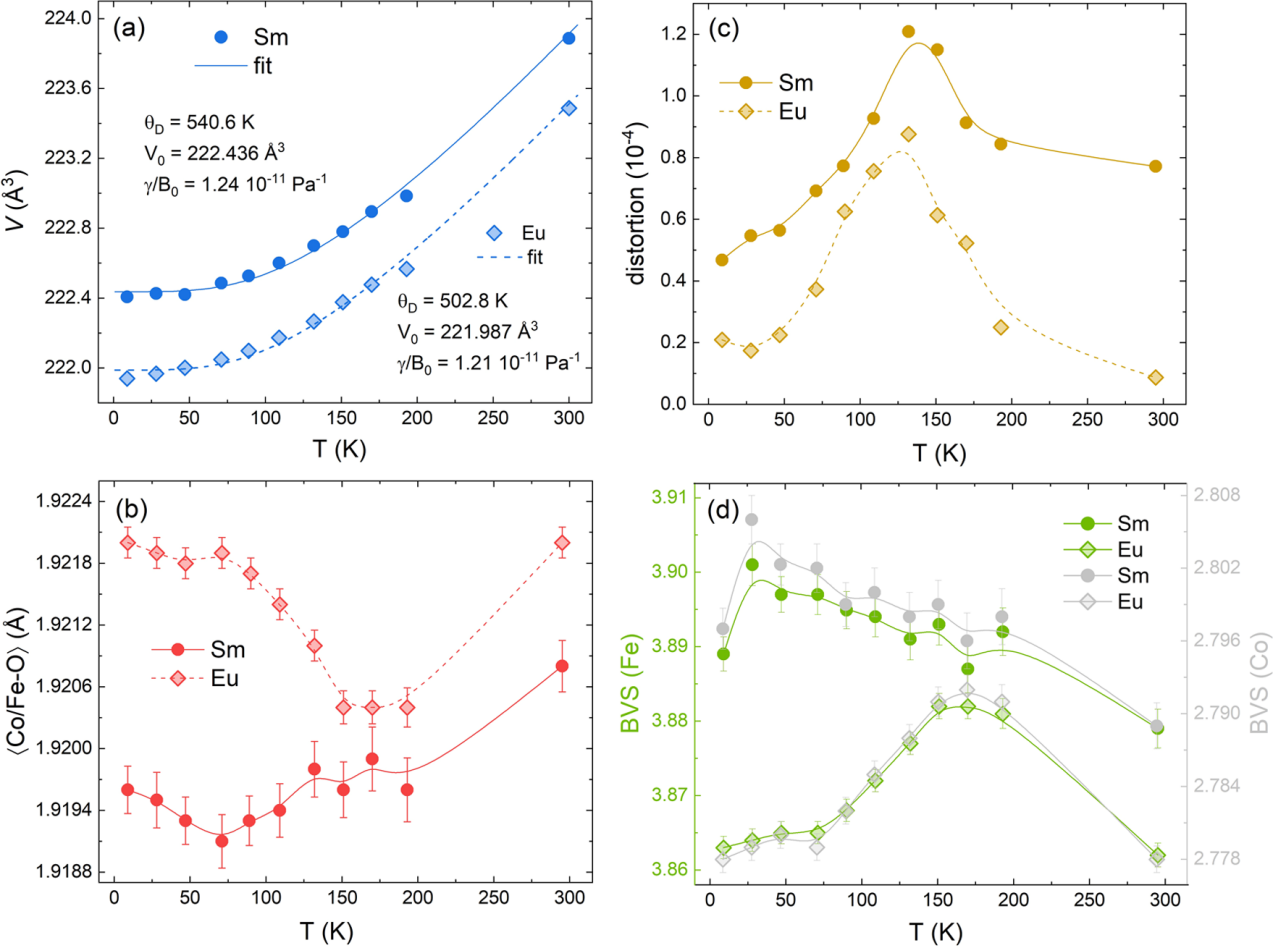
(a) Thermal evolution of the unit-cell volume (*V*), (b) average bond distance (Co/Fe)–O, (c) octahedral distortion, and (d) formal valence of Co and Fe cations [estimated based on the bond valence model (BVS)], extracted from the refined crystal structures for Sm and Eu samples.

The thermal expansion of the unit-cell volume was fitted to the Grüneisen model for the zero-pressure equation of state according to its first-order expansion, i.e.,^45,46^
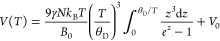
2where *V*_0_ is the 0 K unit-cell volume, θ_D_ is the Debye temperature, *N* is the number of atoms in the unit-cell, *B*_0_ is the isothermal bulk modulus, and γ is the Grüneisen parameter. In Figure 7a, we compare the experimental volume expansion and the best-fit curve using eq 2. The fitting provided *V*_0_ = 222.436 Å^3^, θ_D_ ∼ 541 K, and γ/*B*_0_ = 1.24 × 10^–11^ Pa^–1^ for R = Sm, while for R = Eu we obtained *V*_0_ = 221.987 Å^3^, θ_D_ ∼ 503 K, and γ/*B*_0_ = 1.21 × 10^–11^ Pa^–1^. These Debye temperature values are in good agreement with those reported for GdSrCoFeO_6_,^11^ CaGeO_3_,^47^ and BaZrO_3_^48^, which are likewise derived from the fitting of volume thermal expansion. A comparison can be made to describe the volumetric coefficient of thermal expansion α_V_ = Δ*V*/*V*_*i*_ × Δ*T*,^23^ where *V*_*i*_ is the initial volume and Δ*V* is the volume change corresponding to the temperature change Δ*T*, leading to α_V_ = 1.41 × 10^–5^ K^–1^ (Sm) and α_V_ = 1.54 × 10^–5^ K^–1^ (Eu) in the temperature range 9–193 K. These values are in the same magnitude order of the Pb_2_CoMoO_6_ double perovskite with α_V_ = −1.33 × 10^–5^ K^–1^ in the temperature range of 30–420 K.^23^

### 4.2 Magnetoelastic Coupling

Anomalies in the structural parameters near the magnetic transition temperature are generally associated with the presence of a magnetoelastic coupling^9−11,49^ and require a detailed investigation to understand and clarify this correlation. The R/Sr–O (R = Sm, Eu) and Co/Fe–O bond distances in the crystallographic *a*-, *b*-, and *c*-axis directions were extracted from the refined crystal structures and are displayed in Figure S8. In particular, the ⟨Co/Fe–O⟩ average bond distance (see Figure 7b) revealed that a clear anomalous trend occurred near the magnetic transition temperature for both R = Sm and Eu, such as a slight bond contraction for R = Eu at ∼90 K, which appears to take place at ∼75 K in R = Sm. The previous results are coupled with lattice-parameter variations around 80–90 K, which agree well with the *T*_N_ = 94 (Eu) and 93 K (Sm) magnetic transition temperatures. From these bond distances, the formal valence of Co and Fe cations can be estimated based on the bond valence model (BVS), which accounts for the interdependence between the bond valence and bond length in ionic solids.^50^ The atomic valences of Fe and Co were calculated through the sum of the individual bond valences (*s*_*ij*_), as follows
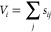
3where the bond valence is the cation–anion interaction and is separated by the distance *d*_*ij*_, i.e.,
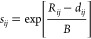
4such that *R*_*ij*_ and *B* are empirical quantities, in which *B* takes values around 0.37 and *R*_*ij*_ can be found in the literature.^51,52^ In Figure 7d, the temperature evolution of the formal Co and Fe valences is shown for both R = Sm and Eu. These results confirm that the valence of Fe is 3+ majority, being a bit higher than nominal, while Co is trivalent but with values essentially smaller than 3+, which agrees very well with XPS results.

Furthermore, the significant variation of (Co/Fe)–O distances between 75 and 150 K results in a maximum lattice distortion (shown in Figure 7c) that appears precisely close to the *T*_peak_ of the observed Δ*S*_M_^max^ values (see Figure 6c,d). In general, the magnetoelastic effect can be manifested when the magnetic ions interact with the crystal lattice structure in response to an external magnetic field, where the magnetic order couples with the lattice distortion and results in a change in the lattice parameters. Recently, Lee et al.^9^ investigated the crystal structure and magnetic structure of La_2_CoIrO_6_ by using neutron diffraction and observed that the symmetric distortion magnitude is strongly correlated with the magnetic moment at temperatures below *T*_C_, suggesting a magnetoelastic coupling by cooperative breathing distortions. In the present case, the anisotropic thermal lattice expansion readjusts the hybridization strengths to induce a spin–orbital–lattice coupling and modulate the long-range ordering of Co/Fe spins from the magnetoelastic effects.^11^ Thus, we attribute the anomalous trends along the average Co/Fe–O bond distance, formal valence, and lattice parameters to the occurrence of magnetoelastic coupling in the RSrCoFeO_6_ (R = Sm, Eu) double perovskites. Therefore, these materials showing magnetoelastic effects can be magnetically tuned through composition variation by interchanging the local atomic arrangement.

### 4.3 Addressing the Magnetic Moments and Frustration

**Figure 8:**
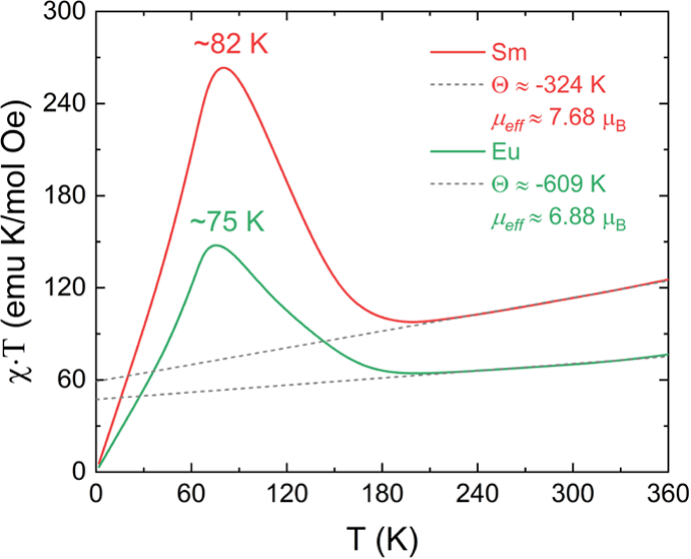
χ·*T*(*T*) curves of RSrCoFeO_6_ (R = Sm, Eu) samples following the C–W law in the high-temperature regime at around 180–360 K.

The high-temperature regions of susceptibility curves for both Sm/Eu samples did not exhibit a typical Curie–Weiss (C–W) behavior, as would be expected for materials with well-localized magnetic moments. Instead, we find a broad and flat plateau in the 200–400 K range (see Figure S9). However, looking at the magnetism of 4*f* and 3*d* orbitals, deviations from the C–W behavior are expected due to the changing magnetic moment associated with the gradual thermal depopulation of excited crystal field states.^53^ A temperature-induced crossover between high-spin and low-spin configurations of transition metals can occur, being associated with decreased lattice vibrations.^53^ These crossovers are principally appreciated most clearly by plotting the χ × *T*(*T*) curves (as in Figure 8). In the high-temperature regime (180–360 K), the curves follow the C–W law, in agreement with the temperature range of rare-earth magnets.^53^ In this case, the term (8χ × *T*)^1/2^ is proportional to the effective magnetic moment (μ_eff_) under the assumption that correlations are negligible, i.e., θ_W_ = 0. Thus, from a linear extrapolation to low temperature, the effective magnetic moments were found to be μ_eff_ = 7.68 (Sm) and 6.88 μ_B_/f.u. (Eu), which is in excellent agreement with the magnetic moment calculated using the atomic compositions estimated from the XPS analysis (detailed in the SI) of μ = 6.77 (Sm) and 6.28 μ_B_/f.u. (Eu). It therefore attests to the occurrence of Co and Fe high-spin configurations in the magnetism of the RSrCoFeO_6_ (R = Sm, Eu) samples.

From the χ·*T*(*T*) plot, the Weiss temperatures were calculated as θ_W_ = −324 K (Sm) and −609 K (Eu), which means that dominant AFM interactions are stronger in the Eu sample. From these values, we estimated the magnetic frustration defined as *f* = |θ_W_|/*T*_N_,^54^ which is an empirical factor to estimate the strength of spin frustration. Typically, magnets with ordering temperatures exhibit *f* > 5, with some materials exceeding *f* = 100^53^. However, nonfrustrated materials have *f* < 5. For instance, several double perovskites containing rare-earth elements as well as Co and/or Fe in their structure present a high magnetic frustration such as *f* ≈ 3.6–4.8 for (La,A)CoNbO_6_ (A = Ca, Sr, and Ba)^55^ and *f* ≈ 8.7–17.8 for Ln_2_LiFeO_6_ (Ln = La, Nd, Sm, and Eu).^56^ Generally, this high frustration comes from the antisite disordered structure and magnetic ions with mixed valences, resulting in short-range magnetic competitions in the crystal structure. On the other hand, we estimated *f* ≈ 0.29 (Sm) and 0.14 (Eu), which indicates that RSrCoFeO_6_ (R = Sm, Eu) double perovskites are nonfrustrated materials, similar to GdSrCoFeO_6_ (*f* ≈ 0.7).^39^ In the case of RSrCoFeO_6_ (R = Sm, Eu) samples, the larger ionic radius of the Sm^3+^ and Eu^3+^ ions compared to the Gd^3+^ ion stabilizes the orthorhombic phase with a less distorted structure, as observed by the larger ⟨Co/Fe–O–Co/Fe⟩ bond angles of 171.9(5)° (Sm) and 168.8(4)° (Eu) compared to 165.9(4)° for GdSrCoFeO_6_.^39^ Consequently, the short-range interactions are weakened between the [Co/Fe]O_6_ octahedra, resulting in less magnetic frustration.

### 4.4 Effectiveness of Magnetic Refrigeration

The effectiveness of magnetic refrigeration was probed through the relative cooling power (RCP) parameter,^57,58^ which is defined by
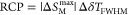
5where δ*T*_fwhm_ is the full width at half-maximum of −Δ*S*_M_(*T*) curves. In Figure 6g, the RCP magnetic field dependence is represented together with their best fit using power-law RCP ≈ μ_0_*H^m^*. These fittings yield *m* = 1.34 ± 0.14 (Sm) and 1.29 ± 0.12 (Eu), which revealed a greater field response compared to that of Δ*S*_M_. We can see that the observed RCP^max^ values (at μ_0_*H* = 0–7 T) of 112.6 J/kg (Sm) and 95.5 J/kg (Eu) are still below some typical compounds, for instance, Gd_5_Ge_2_Si_2_ (240 J/kg),^59^ GdNi_4_Si (322 J/kg),^60^ and TbCo_1.9_Fe_0.1_ (271 J/kg).^61^ Moreover, a comparison of the relative cooling power values, magnetic entropy change, and transition temperature for the investigated samples and other reported double perovskites with Sm/Eu elements on the A-site is listed in Table 1. As noted, the Neel temperature and the maximum magnetic entropy change are smaller when compared to the double perovskites. This is due to Sm/Eu and Sr (nonmagnetic) elements sharing the same AA′-site in a 1:1 ratio, which leads to a large local magnetic disorder and unwanted loss of some Sm/Eu–O–Sm/Eu long-range ferromagnetic interactions, thereby significantly decreasing the Δ*S*_M_. Particularly, the smaller Δ*S*_M_ compared to GdSrCoFeO_6_ (∼13 J/kg K)^39^ is due to the peculiar feature of Gd in presenting a large spin ground state with zero orbital moment (*S* = 7/2 and *L* = 0, i.e., *J* = 7/2) and the spherically symmetric ground state of ^8^*S*_7/2_, which provides the largest entropy per single ion at low temperatures,^62^ unlike Sm and Eu. Furthermore, the strong lattice reorganization resulting from magnetoelastic coupling reinforces the magnetocaloric effect in GdSrCoFeO_6_ at ∼8 K, which in the case of RSrCoFeO_6_ (R = Sm, Eu) is also similarly observed around *T*_N_, but in a much smaller magnitude. In general, the shape and temperature of the magnetic entropy change occurrence are related to the competition of short-range magnetic interactions between the Co/Fe and/or rare-earth ion lattice in the system, which can be intensified by changes in the bond length and the lattice reorganization upon cooling. On the other hand, specifically, the RCP of SmSrCoFeO_6_ is in the same order of magnitude as Eu_2_CoMnO_6_ due to its wider δ*T*_fwhm_. Lastly, the uniform distribution of −Δ*S*_M_(*T*) curves for the RSrCoFeO_6_ (R = Sm,Eu) samples is an interesting feature desirable for ideal magnetic cooling cycles in magnetic refrigerators, making them interesting in this regard.

**Table 1:** Comparison of Δ*S*_M_^max^ and RCP Values for the RSrCoFeO_6_ (R = Sm, Eu) Samples and Other Double Perovskites Containing Sm or Eu on the A-Site

compound	*T*_C_/*T*_N_ (K)	μ_*0*_Δ*H* (T)	Δ*S*_M_^max^ (J/kg K)	RCP (J/kg)	reference
SmSrCoFeO_6_	93	0–7	0.87	112.6	this work
EuSrCoFeO_6_	84	0–7	0.87	95.5	this work
Eu_2_NiMnO_6_	145	0–7	4.0	241.5	(63)
Sm_2_CoMnO_6_	123	0–6	1.4		(44)
Eu_2_CoMnO_6_	123	0–6	3.3		(64)
Eu_2_NiMnO_6_	143	0–5	3.2	150	(2)
EuTbCoMnO_6_	113	0–5	2.3		(65)

## 5 Conclusions

In summary, we systematically studied the structural, electronic, magnetic, and magnetocaloric properties of the new RSrCoFeO_6_ (R = Sm, Eu) double perovskites synthesized by the solid-state reaction process. The structural and morphological analyses reveal that the samples crystallize in a typical *Pnma* (#62) orthorhombic structure with irregularly shaped grains and nonuniform size distribution. Magnetic characterization suggests the existence of long-range AFM order with a second-order phase transition to the paramagnetic state at the Néel temperatures of *T*_N_ ≈ 93 K (Sm) and 84 K (Eu). From extrapolation of χ × *T*(*T*) curves, the effective magnetic moments were found to be μ_eff_ = 7.68 (Sm) and 6.88 μ_B_/f.u. (Eu), which is in agreement with those calculated from the analysis of XPS data [μ = 6.77 (Sm) and 6.28 μ_B_/f.u. (Eu)]. This indicated that the magnetism of the samples predominantly results from spin–orbit coupling for Sm^3+^ and Eu^3+^, whereas spin-only interactions occur for Co^2+^/Co^3+^ and Fe^3+^/Fe^4+^ in the high-spin states. The magnetic entropy change investigation showed a conventional magnetocaloric effect with well-defined positive peaks around *T*_N_, yielding Δ*S*_M_^max^ ≈ 0.87 J/kg K (for both samples) and relative cooling powers of RCP ≈ 112.6 J/kg (Sm) and 95.5 J/kg (Eu). In particular, the anomalies along the average Co/Fe–O bond distance, formal valence, octahedral distortion, as well as an anisotropic lattice expansion revealed by temperature-dependent SXRD data coincide well with the magnetic transition temperatures *T*_N_, which we attributed to the occurrence of magnetoelastic coupling in the RSrCoFeO_6_ (R = Sm, Eu) double perovskites. The magnetoelastic coupling is responsible for readjusting the hybridization strengths, inducing a spin–orbital–lattice coupling that modulates the long-range magnetic ordering in these systems. Therefore, the results presented in this work can pave a new road for fine-tuning the magnetic properties and, especially, for understating the mechanism of the magnetocaloric effect in double perovskites based on (Co, Fe) at the B-site.

## Supporting Information Available

Additional experimental details, materials, and methods, including structural parameters, details on SXRD patterns, EDX analyses, XPS data, Arrott plots, and inverse magnetic susceptibility (χ^–1^) data (PDF)
