# Supplementary material for: Magnetoelastic Coupling Evidence by Anisotropic Crossed Thermal Expansion in Magnetocaloric RSrCoFeO6 (R = Sm, Eu) Double Perovskites
Source: Inorg Chem. 2024 Apr 1;63(15):7007–18. doi: 10.1021/acs.inorgchem.4c00594 (PMC11022179; doi:10.1021/acs.inorgchem.4c00594)
Supplement: Supplementary file 1 — ic4c00594_si_001.pdf [file ic4c00594_si_001.pdf]

Supplementary Information

Magnetoelastic coupling evidence by anisotropic crossed thermal expansion in the magnetocaloric *R*SrCoFeO$_6$ (*R* = Sm, Eu) double perovskites

Romualdo S. Silva Jr.[1*], Joao E. Rodrigues[2,3], Javier Gainza[1], Federico Serrano-Sánchez[1], Lidia Martínez[1], Yves Huttel[1], José Luis Martínez[1], José Antonio Alonso[1*]

[1] *Instituto de Ciencia de Materiales de Madrid (ICMM), CSIC, E-28049 Madrid, Spain.*

[2] *European Synchrotron Radiation Facility (ESRF), 71 Avenue des Martyrs, 38000 Grenoble, France.*

[3] *CELLS-ALBA Synchrotron, E-08290 Barcelona, Spain.*

[*] Corresponding author: *ja.alonso@icmm.csic.es, romualdo.silva@csic.es.*

Table S1: Structural parameters of the Sm and Eu samples at room temperature obtained through Rietveld refinement from SXRD data.

**$SmSrCoFeO_6$**					
**atom**	**Wyckoff site**	***x***	***y***	***z***	**B_{iso}**
Sm/Sr	4*c*	0.50953(1)	0.25	0.49516(2)	1.095(8)
Co/Fe	*4b*	0.50	0.00	0.00	0.562(1)
O1	4*c*	-0.00192(1)	0.25	0.51037(1)	4.184(1)
O2	8*d*	0.25990(2)	0.02513(5)	0.73546(1)	1.987(9)
**lattice parameters**		**reliability factors**		**average bonds and angles**	
a (Å)	5.3984(7)	R_p (%)	7.69	⟨Sm/Sr − O1⟩ (Å)	2.709(5)
b (Å)	7.6334(9)	R_{wp} (%)	11.4	⟨Sm/Sr − O2⟩ (Å)	2.639(7)
c (Å)	5.4329(3)	R_{exp} (%)	4.86	⟨Co/Fe − O1⟩ (Å)	1.909(2)
V ($Å^3$)	223.88(7)	R_{Bragg} (%)	5.95	⟨Co/Fe − O2⟩ (Å)	1.927(4)
$\alpha=\beta=\gamma$ (°)	90	R_f (%)	8.88	⟨Co/Fe − O1 − Co/Fe⟩ (°)	176.6(5)
ρ (g/cm^3)	6.658	χ^2	5.52	⟨Co/Fe − O2 − Co/Fe⟩ (°)	167.3(3)
**$EuSrCoFeO_6$**					
**atom**	**Wyckoff site**	***x***	***y***	***z***	**B_{iso}**
Eu/Sr	4*c*	0.51308(0)	0.25	0.49555(1)	1.068(8)
Co/Fe	*4b*	0.50	0.00	0.00	0.489(7)
O1	4*c*	-0.00134(9)	0.25	0.52841(1)	5.325(2)
O2	8*d*	0.26598(9)	0.02386(4)	0.73355(8)	1.279(6)
**lattice parameters**		**reliability factors**		**average bonds and angles**	
a (Å)	5.3985(1)	R_p (%)	6.87	⟨Eu/Sr − O1⟩ (Å)	2.709(8)
b (Å)	7.6329(8)	R_{wp} (%)	9.67	⟨Eu/Sr − O2⟩ (Å)	2.630(1)
c (Å)	5.4235(7)	R_{exp} (%)	1.30	⟨Co/Fe − O1⟩ (Å)	1.914(6)
V ($Å^3$)	223.48(8)	R_{Bragg} (%)	4.39	⟨Co/Fe − O2⟩ (Å)	1.926(0)
$\alpha=\beta=\gamma$ (°)	90	R_f (%)	5.73	⟨Co/Fe − O1 − Co/Fe⟩ (°)	170.8(4)
ρ (g/cm^3)	6.693	χ^2	5.53	⟨Co/Fe − O2 − Co/Fe⟩ (°)	166.8(3)

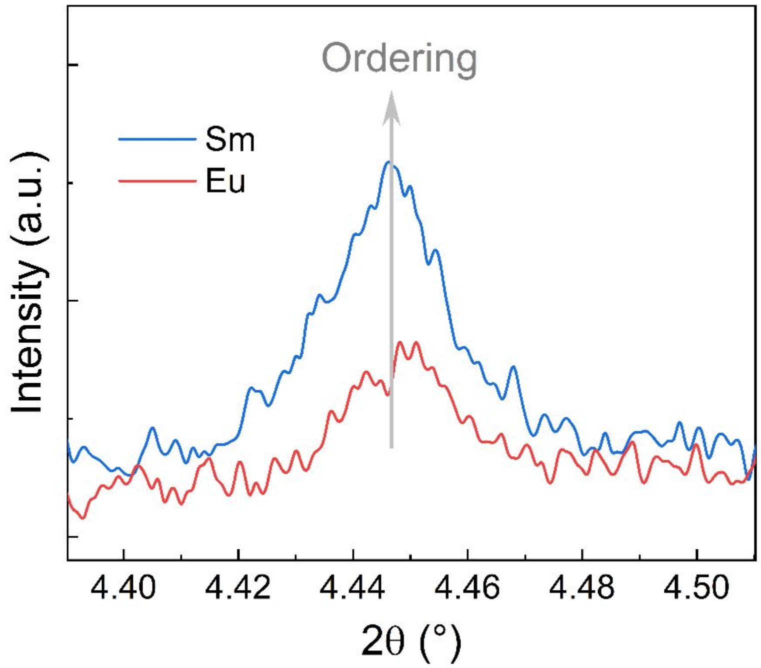

Figure S1: High-resolution synchrotron X-ray diffraction (SXRD) data between ~ 4.35−4.55° range of the RSrCoFeO$_6$ (R = Sm, Eu) samples collected at room temperature.

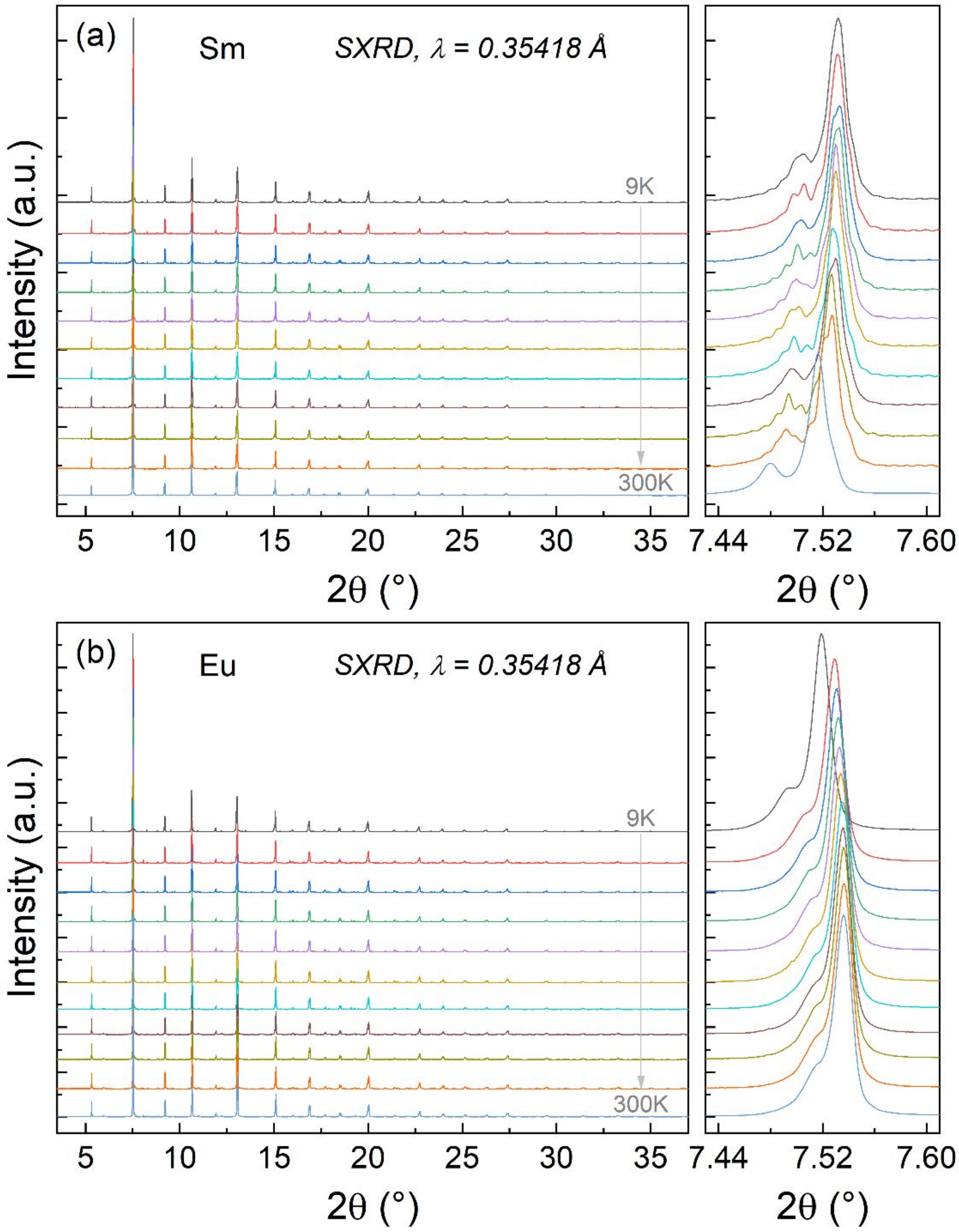

Figure S2: SXRD temperature-dependent in the 9-300 K range for the (a) $SmSrCoFeO_6$ and (b) $EuSrCoFeO_6$ samples.

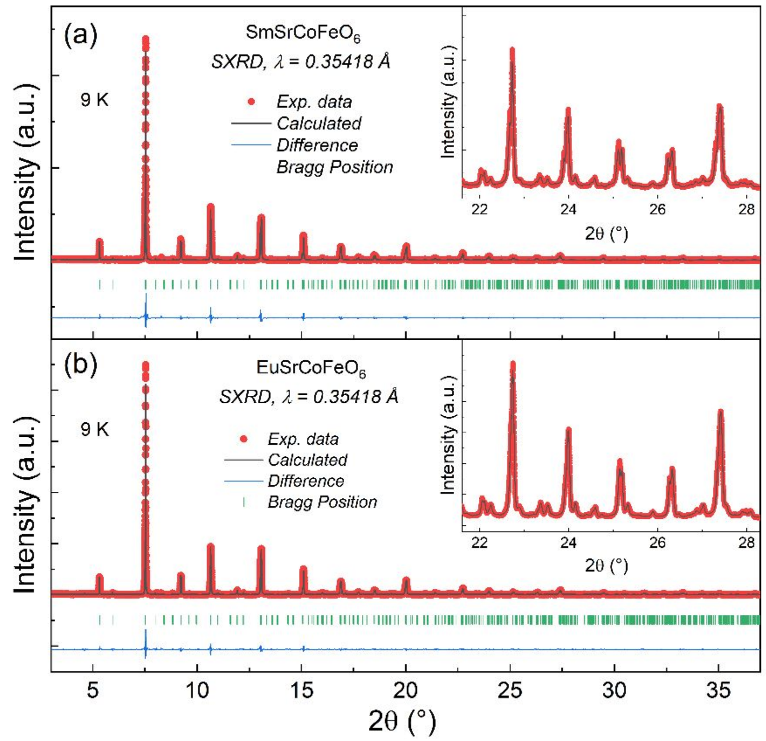

Figure S3: Rietveld refinement of SXRD data for the (a) $SmSrCoFeO_6$ and (b) $EuSrCoFeO_6$ samples at 9 K.

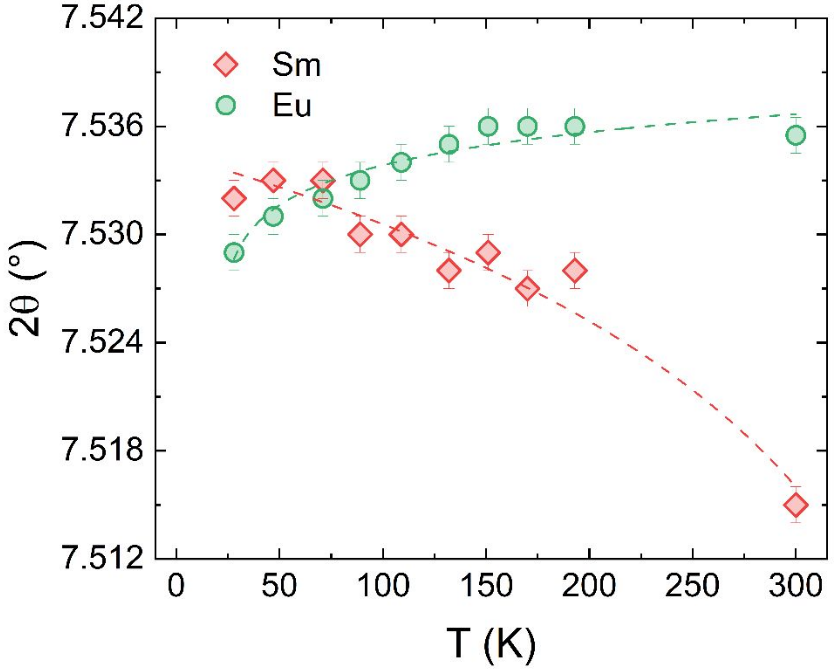

Figure S4: Main SXRD peak position temperature-dependent of the RSrCoFeO$_6$ (R = Sm, Eu) samples.

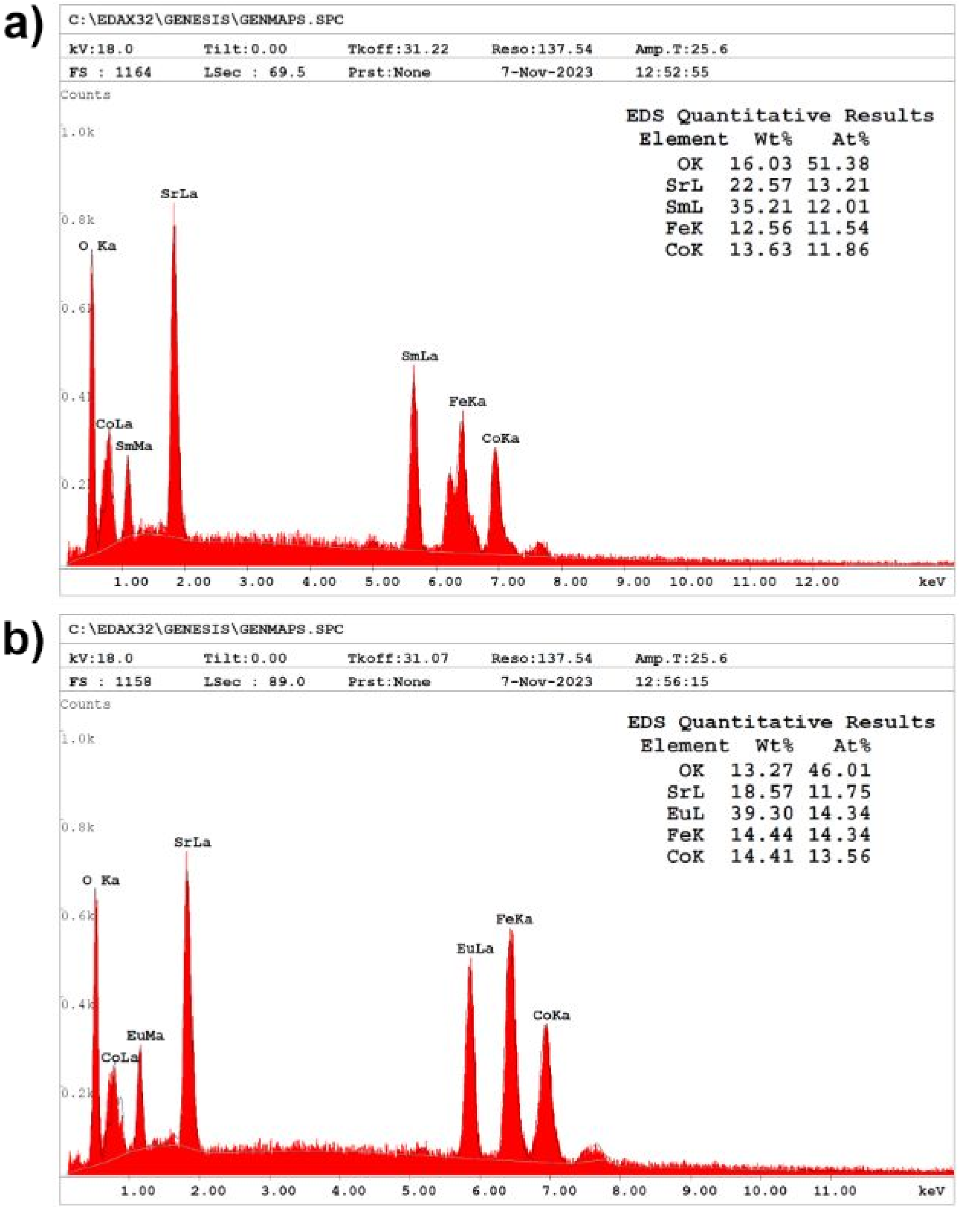

Figure S5: EDX analyses with quantitative results of the elements for the (a) $SmSrCoFeO_6$ and (b) $EuSrCoFeO_6$ samples.

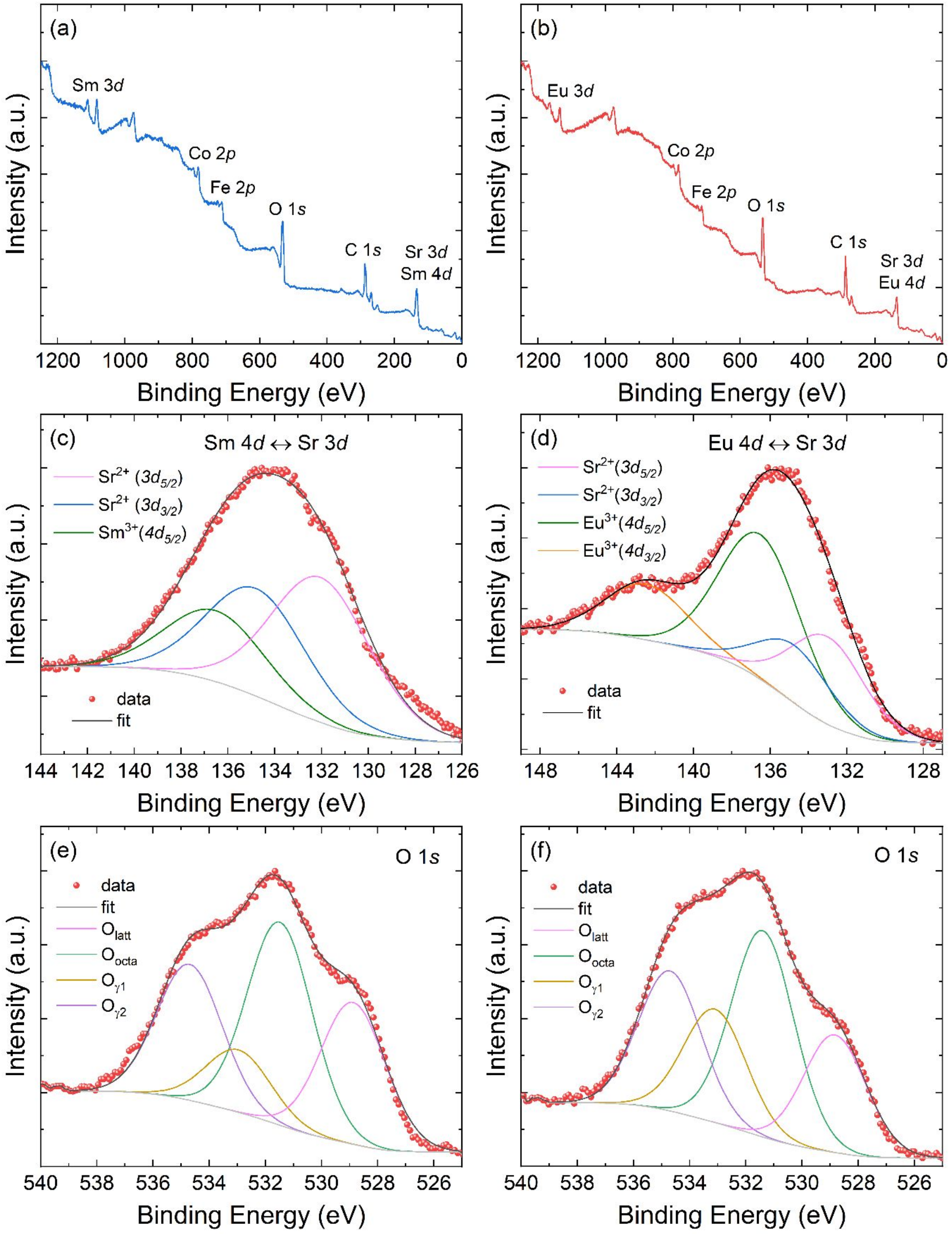

Figure S6: XPS survey and core level spectra of Sr 3*d*-Sm 4*d*, Sr 3*d*-Eu 4*d*, and O 1*s* for the $SmSrCoFeO_6$ (a,c,e) and $EuSrCoFeO_6$ (b,d,f) samples, respectively. The carbon and part of the oxygen present in the

surveys is caused by ex-situ analysis.

The quantitative analysis of the $SmSrCoFeO_6$ and $EuSrCoFeO_6$ samples was extracted from the wide scan energy spectra and the results are given in Table S2.

Table S2: Quantitative analysis of the elements for the $SmSrCoFeO_6$ and $EuSrCoFO_6$ samples from XPS spectra.

	**$SmSrCoFeO_6$**	**$EuSrCoFeO_6$**
	**Composition / at.%**	
**C 1*s***	52.4	55.2
**Sm 3*d***	1.3	-
**Eu 3*d***	-	0.7
**Sr 3*d***	5.2	4.8
**Co 2*p***	2.4	2.6
**Fe 2*p***	2.5	2.2
**O 1*s***	36.2	34.5

Table S3: Normalized quantitative analysis of the mixed valences of Co and Fe, removing the contribution of carbon and the Auger peaks of Co and Fe for each sample.

	**$SmSrCoFeO_6$**	**$EuSrCoFeO_6$**
	**Composition / at.%**	
**Co^{2+}**	34.91	32.64
**Co^{3+}**	65.09	67.36
**Fe^{3+}**	72.85	49.01
**Fe^{4+}**	27.14	50.99

From the quantitative analysis (at.%) of Co^{2+}/Co^{3+} anf Fe^{3+}/Fe^{4+} elements shown in Table S3, we have calculated the magnetic moment of $SmSrCoFeO_6$ and $EuSrCoFeO_6$ samples respectively as follows:

$$\mu \approx \left[(\mu_{Sm^{3+}})^2 + 0.35(\mu_{Co^{2+}})^2 + 0.65(\mu_{Co^{3+}})^2 + 0.73(\mu_{Fe^{3+}})^2 + 0.27(\mu_{Fe^{4+}})^2\right]^{1/2}\mu_B$$

$$\mu \approx \left[(\mu_{Eu^{3+}})^2 + 0.33(\mu_{Co^{2+}})^2 + 0.67(\mu_{Co^{3+}})^2 + 0.49(\mu_{Fe^{3+}})^2 + 0.51(\mu_{Fe^{4+}})^2\right]^{1/2}\mu_B$$

considering the PM moments with spin-orbit coupling in the high-spin (HS) states for Sm^{3+} (J = 5/2, g_J = 2/7, $\mu = 0.85\ \mu_B$) and Eu^{3+} (J = 0, g_J = 0, $\mu = 0\ \mu_B$), whereas using spin-only interactions

for Co^{2+} (S = 3/2, $\mu = 3.87\ \mu_B$), Co^{3+} (S = 2, $\mu = 4.9\ \mu_B$), Fe^{3+} (S = 5/2, $\mu = 5.92\ \mu_B$), and Fe^{4+} (S = 1, $\mu = 2.82\ \mu_B$) [1], which yielding μ = 6.77 (Sm) and 6.28 μ_B/f.u. (Eu).

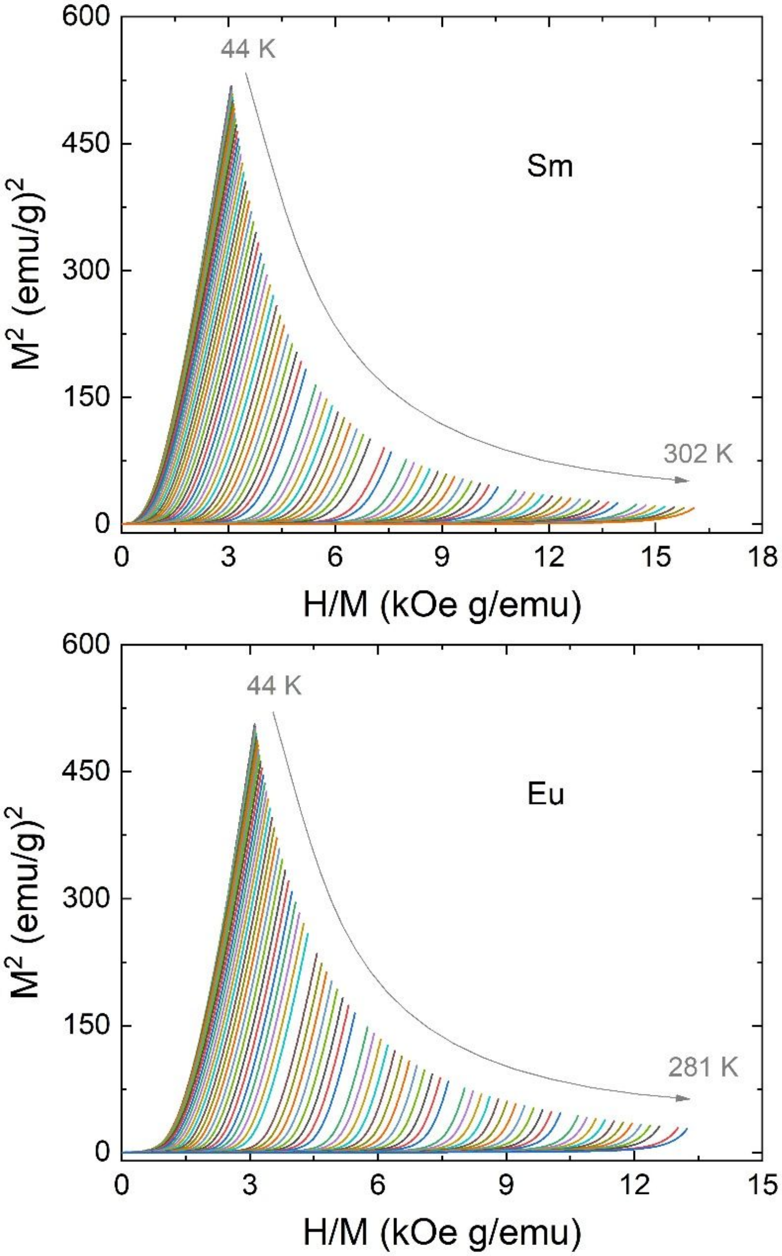

Figure S7: Arrott plots (M^2 vs H/M) of the RSrCoFeO$_6$ (R = Sm, Eu) samples constructed from the $M(H)$

data in the 44-302 K temperature range.

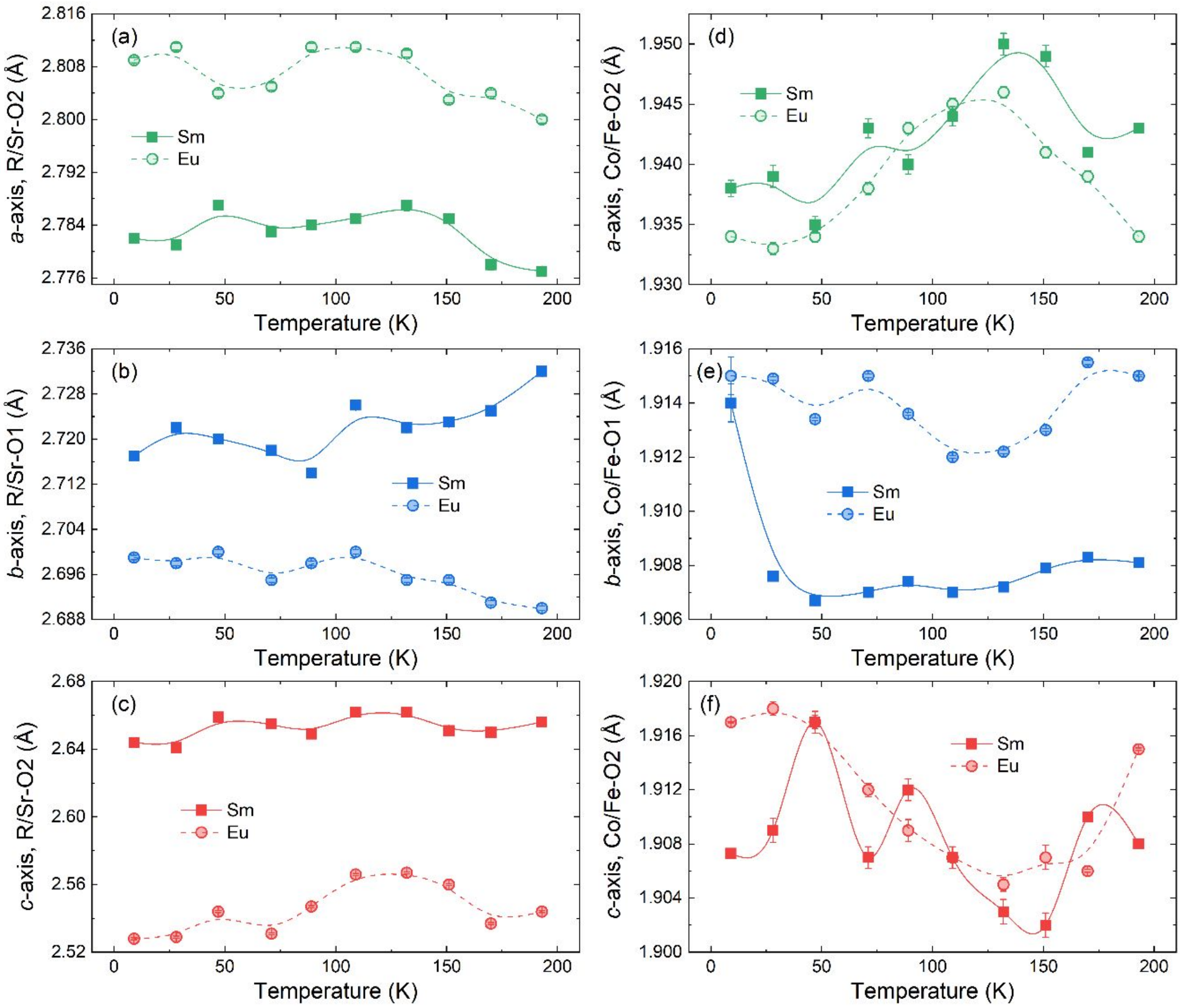

Figure S8: (a-c) R/Sr-O and (d-f) Co/Fe-O distances in the crystallographic *a*-,*b*-, and *c*-axis direction, respectively.

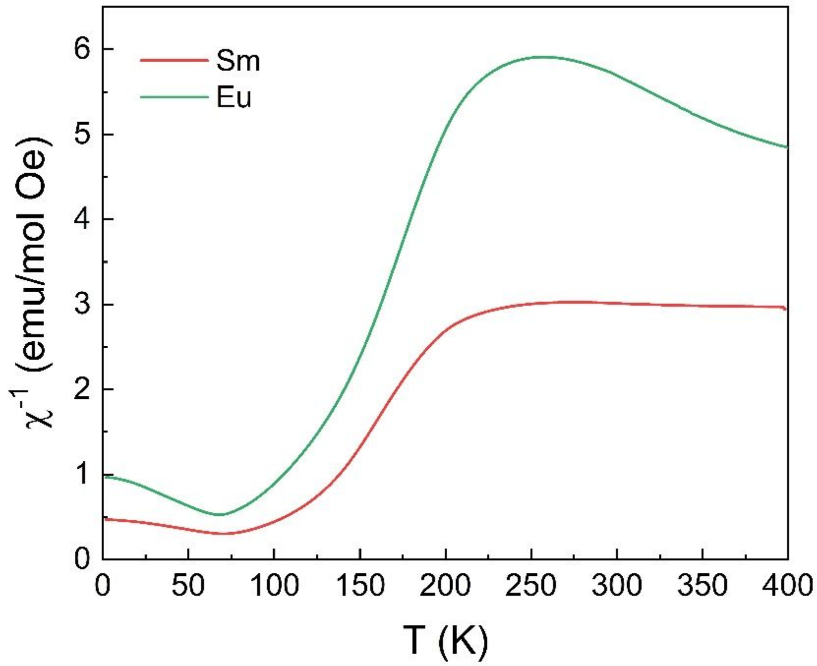

Figure S9: Inverse magnetic susceptibility (χ^{-1}) as a function of temperature for *R*$SrCoFeO_6$ (*R* = Sm, Eu) samples.

(1) Mugiraneza, S.; Hallas, A. M. Tutorial: A Beginner's Guide to Interpreting Magnetic Susceptibility Data with the Curie-Weiss Law. *Commun. Phys.* **2022**, *5* (1). https://doi.org/10.1038/s42005-022-00853-y.
